# PON-Fold: Prediction of Substitutions Affecting Protein Folding Rate

**DOI:** 10.3390/ijms241613023

**Published:** 2023-08-21

**Authors:** Yang Yang, Zhang Chong, Mauno Vihinen

**Affiliations:** 1 School of Computer Science and Technology, Soochow University, Suzhou 215006, China; yyang@suda.edu.cn (Y.Y.); 20205227090@stu.suda.edu.cn (Z.C.); 2 Collaborative Innovation Center of Novel Software Technology and Industrialization, Nanjing 210000, China; 3 Department of Experimental Medical Science, Lund University, BMC B13, SE-221 84 Lund, Sweden

**Keywords:** protein folding, protein unfolding, machine learning, variation interpretation, amino acid substitution, folding rate

## Abstract

Most proteins fold into characteristic three-dimensional structures. The rate of folding and unfolding varies widely and can be affected by variations in proteins. We developed a novel machine-learning-based method for the prediction of the folding rate effects of amino acid substitutions in two-state folding proteins. We collected a data set of experimentally defined folding rates for variants and used them to train a gradient boosting algorithm starting with 1161 features. Two predictors were designed. The three-class classifier had, in blind tests, specificity and sensitivity ranging from 0.324 to 0.419 and from 0.256 to 0.451, respectively. The other tool was a regression predictor that showed a Pearson correlation coefficient of 0.525. The error measures, mean absolute error and mean squared error, were 0.581 and 0.603, respectively. One of the previously presented tools could be used for comparison with the blind test data set, our method called PON-Fold showed superior performance on all used measures. The applicability of the tool was tested by predicting all possible substitutions in a protein domain. Predictions for different conformations of proteins, open and closed forms of a protein kinase, and apo and holo forms of an enzyme indicated that the choice of the structure had a large impact on the outcome. PON-Fold is freely available.

## 1. Introduction

During protein folding, the characteristic three-dimensional structure is obtained. Folding can be co-translational during translation or happen after synthesis. Folding rate differences between proteins vary by five orders of magnitude [1]. Interestingly, the unfolding rates have a substantially wider range, up to 10 orders of magnitude [2].

Details for protein folding are available from PFDB [3] and ACPro [4] databases. Several methods based on different principles and algorithms predict protein folding rates, for a review and benchmarking study (see [5]). These tools estimate the folding of entire proteins and the performance varies widely, from very poor to good.

Variations, such as amino acid substitutions, can affect many protein properties, including stability, activity, solubility, structure, etc. Variants can also affect protein folding rates, usually in a deleterious way. In human muscle acylphosphatase substitutions, such as Y25F and V68A, have a major effect on the folding rate [6]. The available folding rate values for variants originate mainly from a single large study of 806 variants in 24 proteins [7]. Folding and unfolding rates in Rop, a four-helix-bundle protein, vary over four orders of magnitude [8].

It is more difficult to predict the effects of variations on protein folding than the folding rates of proteins. Previously, one research group used the same or slightly increased data sets to develop several predictors. The algorithms they applied include quadratic regression in FORA [9] and FREEDOM [10], rule-based decision tree in KD-FREEDOM [11], multiple linear regression in Folding RaCe [12], and amino acid properties and multiple linear regression in Unfolding RaCe [2]. In addition, an application based on residue-level coevolutionary networks has been presented [13]. A related problem is an estimation of the effects of variations on protein folding free energy and other properties. A free-energy approach utilizing a modified Molecular Mechanics Generalized Born (MMGB) is an example [14].

Protein folding and solubility are closely connected [5]. There are different fates for proteins regarding their solvent interaction (see [15]). Soluble proteins can precipitate, misfolded proteins can refold spontaneously or with the help of chaperones, or become sequestered or aggregated, when having irreversible structural alterations. Previously, we developed reliable machine learning (ML) predictors called PON-Sol [16] and PON-Sol2 [17] for predicting the solubility effects of amino acid substitutions.

Here, we present an ML-based approach for folding rate effect predictions due to amino acid substitutions. The method was implemented with a gradient boosting algorithm and was trained with a large data set obtained from the literature. A comparison with a previous tool indicated that PON-Fold, our tool, had superior performance. We used the new method to predict all possible substitutions in a widely studied protein and correlated the results to pathogenicity predictions. Then, the method was used to study the effects in different conformations of proteins in open and closed forms of a protein kinase and in apo and holo forms of an enzyme. Protein conformation has a substantial effect on the prediction outcome; therefore, the choice of the structure is a crucial step.

## 2. Results and Discussion

### 2.1. Selection of Data Sets

Data for folding affecting variants were collected from previous predictor articles and the literature. In total, there were 952 variants (Table 1) in 29 two-state proteins (30 PDB entries) (Supplementary Table S1). Most of the folding rate measurements were performed with a stopped-flow fluorimeter, some of them also with a continuous flow fluorimeter, temperature jump fluorimeter, or stopped-flow circular dichroism (CD). Folding effects were divided into three categories: increasing, decreasing, and not affecting the folding rate. As there is not an agreed definition of no-effect variants, we optimized the cutoff for the distribution of cases into three categories so that there were substantial numbers of cases in all three categories. The threshold was set to ±0.15 s^−1^. The data items were then distributed to training and blind test data sets. As a guiding principle, all variants in a protein and position were kept together either in the training or test data set. This was conducted to avoid any bias in training and performance assessment. The data set is the largest collection of folding rate-affecting variants ever used.

The distribution of the amino acid substitutions in the data set is shown in Supplementary Table S2. The data set is biased for some residues. Alanine is the most common variant residue accounting for more than half of the cases. This is because alanine scanning mutagenesis has been widely used to study the effects of amino acid side chains. Other frequently substituting residues are phenylalanine and valine. For some amino acid substitutions, there are no cases at all. There are variations for all amino acid types, although the numbers differ greatly. Leucine, valine, and histidine are the most frequently altered amino acid types.

The training set contained 520 folding-rate-decreasing, 106 increasing, and 136 variants with no effect, totaling 762 variants. The blind test set consisted of 190 variants, of which 133 decreased the folding rate, 39 had no effect, and 18 increased the folding rate.

**Figure 1:**
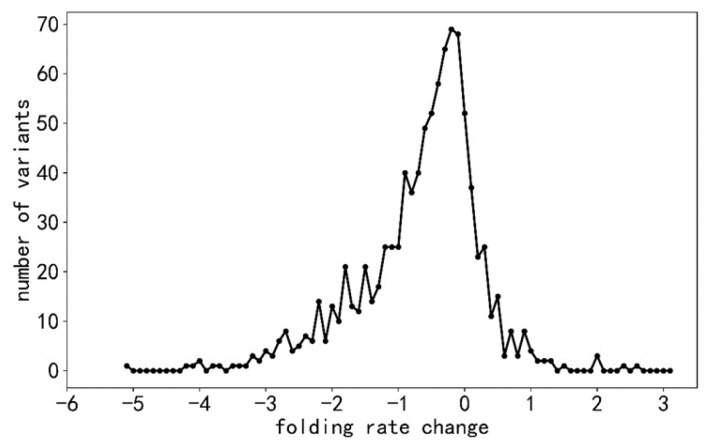
Distribution of folding rate changes for variants.

The folding rate scores for all variants are shown in Figure 1. They follow quite well the normal distribution. The data are biased towards stability-decreasing variants and have wider distribution on that side. Variants that reduce or delete a protein property are more frequent also for pathogenicity, stability, solubility, and activity than those that increase the property. Folding-rate-increasing variants are substantially rarer than those in the two other categories, as shown in Table 1.

### 2.2. Three-Class Classifier

The collected data items were divided into three categories. Since the variants had numerical folding rate values, we were able to develop both a classifier and a regression predictor.

**Figure 2:**
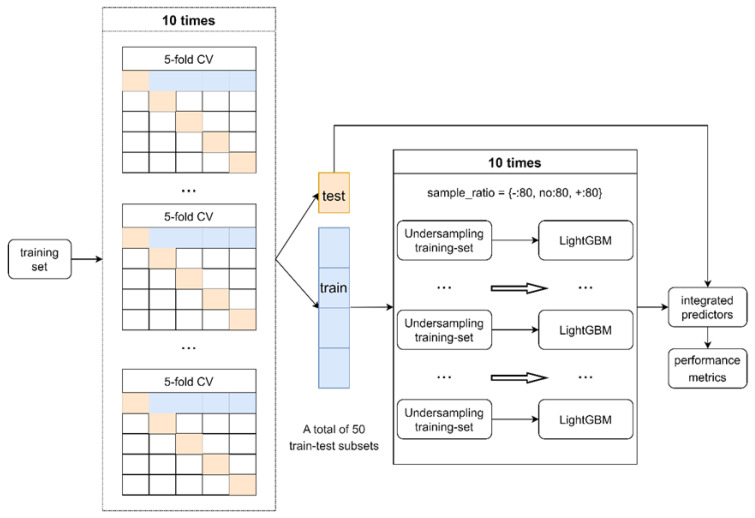
Flowchart of a classification model.

As the data set was unbalanced for the number of cases in the three categories, we used an iterative process to take benefit of all the cases. We generated a total of 50 random partitions of training cases with equal distribution in the three categories (Figure 2). For each subset, we trained a predictor with LightGBM (Microsoft Corporation, Redmond, WA, USA) and selected the features based on all 50 predictors.

**Figure 3:**
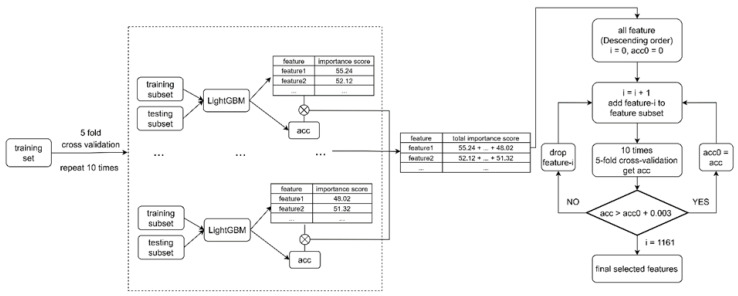
Flowchart of feature selection.

Five-fold cross-validation was used 10 times to estimate the performance of predictions. The flowchart for the feature selection for the classification predictor is shown in Figure 3. Predictors were trained with LightGBM by dropping one feature at a time in each iterative step. Feature importance was ranked over all the predictors to obtain the final list of significant features. LightGBM was chosen as the algorithm since it has proven reliable and the best algorithm in our recent variant interpretation tools, including the PON-All generic pathogenicity predictor [18], PON-Sol2 variant severity predictor [17], and ProTstab protein cellular stability predictor [19].

Feature selection for three-class classification showed the best performance with 31 features (Table 2). None of the selected features had very high scores, and much more important scores were seen, e.g., when training PON-All. The conservation score (C-score) reflects the structural and functional importance of the variant position, which is the most important feature followed by relative position (rp) and relative solvent accessibility (rsa) for the extent of accessibility of the original amino acid. We started with a large number of features of many types. This was important since the selected features represent all the feature categories. There are several amino acid propensities, neighbor features, amino acid substitution types, potentials calculated in different ways, and proportions of amino acid types. The fact that none of the features has a very high score originates from the difficult prediction task.

The procedure for training the predictor is shown in Figure 3. Five-fold CV was repeated 10 times by using under-sampled training data sets with equal numbers of variants in the three categories. Each set contained 80 folding-rate-increasing, decreasing, and non-affecting variants. The results are shown in Table 3. We followed the guidelines for reporting predictor performance [20,21] and provide the full set of measures.

In the CV, the specificity ranges from 0.493 to 0.591 and the sensitivity ranges from 0.527 to 0.540 for different types of variants (Table 3). The corresponding scores in the blind test set are from 0.324 to 0.419 and from 0.256 to 0.451, i.e., somewhat lower for all types of variants (Table 3). The values for F1 behave the same way. This may indicate that the use of additional cases could significantly improve performance. The problem is that such data are not often published.

The other scores are also lower for the blind test set. The performance measures are somewhat better for 31 selected features than for all the features. The measures are typically somewhat better when trained on all the features than on the selected features in the blind test set; however, the differences are not large.

Accuracy, macro-F1, and GC2 were calculated over the entire data. On CV, the performance was clearly better when using the selected features. In the case of blind test data, somewhat better results were obtained with all the features. As the differences are not large, and the use of all the features introduces the so-called curse of dimensionality, especially since we have substantially more features than cases, it was preferable to use a smaller feature set. For extensive discussion on the representativeness of training data sets (see [22]). Small data sets cannot cover the entire space of feature combinations.

### 2.3. Regression Predictor

As another application, we trained a regression predictor for the value of folding rate change. The feature selection was performed similar to that described above. A total of 21 features were identified as the most informative ones (see Table 4). There is some overlap with the features in Table 2: nine (43%) of the features are the same. The common features include the three most important features: relative solvent accessibility, C-score, and the relative position of the variant in the sequence. In addition, a quasichemical potential, a distance-dependent potential, a principal component, and transfer free energy were shared. Several of the amino acid neighborhood features are shared including those for aromatic tyrosine and phenylalanine, charged aspartate, and positively charged residues. The remaining features are for some potentials, amino acid types, and others.

The full set of features and the 21 selected features were used to test the performance in 10-time five-fold CV (Table 5). We used four measures to address the performance. All the scores are better when using 21 selected features instead of all the features. The scores are good, and PCC is 0.525. The error measures, MAE and MSE, are 0.581 and 0.603, respectively. R2 (0.255) is substantially better for the selected features. This measure, the coefficient of determination, provides a measure of how well the observed cases match with the model. It is calculated based on the proportion of total variation in cases explained by the predictor.

PON-Fold has good performance considering the difficult prediction task and limited and biased data set. Some variation types are scarce or missing from the training data and affect the performance of the method.

### 2.4. Blind Test Performance

Several methods have been presented for variant effect calculation for folding rate change (see the Introduction). However, only one of these, Folding RaCe [12], was available for comparison. The other tools are either not available or do not facilitate large-scale prediction. FoldingRaCe uses relative solvent accessibility, secondary structural information, and position in the sequence as features. It constructs sub-models by multiple linear regression to predict the folding rate change. We compared the performance of our tool to that of Folding RaCe on the blind test set. PON-Fold has better performance according to all metrics (Table 6). For example, the Pearson correlation coefficient is better by 16 percentage points. The error measures, MAE and MSE, are substantially better for PON-Fold, both being well under 1 s^−1^. In conclusion, PON-Fold has substantially better performance. It was trained on 752 variants, whereas the FoldingRace is based on 790 variants. Since the numbers of cases are almost identical for the two methods, the differences in the performance are due to a better representation of the features relevant to the folding rate in PON-Fold.

**Figure 4:**
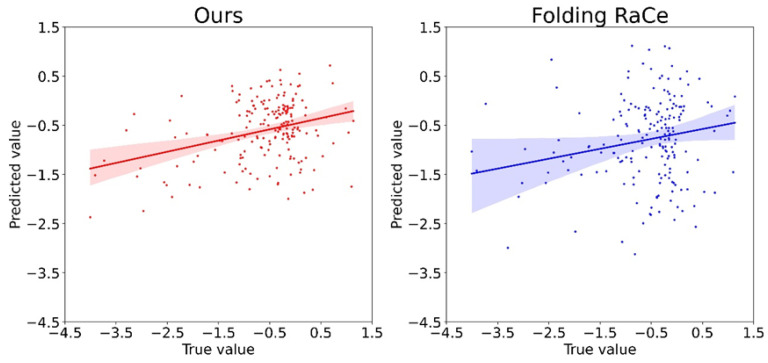
Correlation of true and predicted values. The shaded areas represent 95% confidence intervals.

Figure 4 shows the distribution of the true and predicted values for PON-Fold and Folding RaCe. The region for a 95% confidence interval is substantially narrower for our method and is indicated by the error measures. The range of distribution is very narrow in the case of PON-Fold, which is indicative of good performance. There is still room for improvements, which could be achieved with the extended data set. This is important as proteins are widely different and to obtain generalizable features larger numbers would be necessary. Unfortunately, such data sets are not frequently determined.

### 2.5. PON-Fold Application to Domain-Wide Analysis of Folding Effects

**Figure 5:**
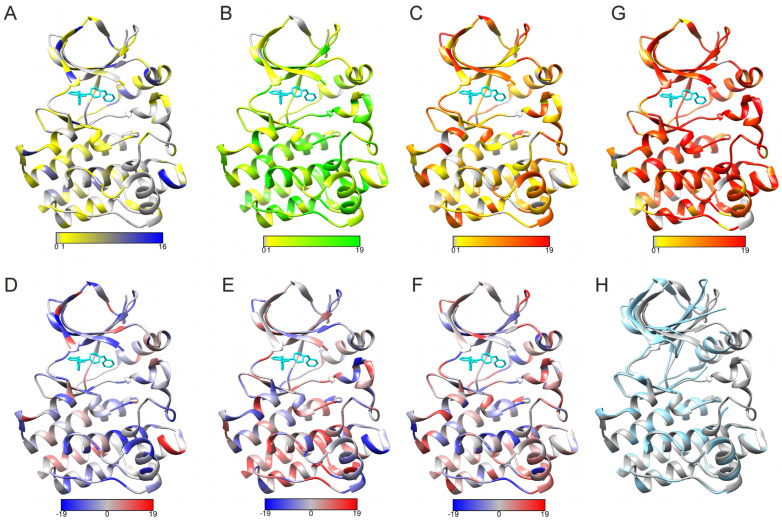
Predicted folding effects indicated on BTK kinase domain in closed (PDB code 3gen) and open (3k54) structures. The range of (**A**) folding decreasing, (**B**) no effect, and (**C**) folding increasing variants are color-coded. The scales below the structures show the numbers of each predicted effect due to the variations. The differences in the numbers of (**D**) folding decreasing, (**E**) no effect, and (**F**) folding increasing variants between the open and closed conformations. The scales below the structures show the differences in numbers of each predicted effect due to the variations, closed enzyme vs. open enzyme. (**G**) Variants predicted to cause disease, X-linked agammaglobulinemia, in BTK kinase domain (3gen). Predictions were made with PON-P2 program. The scale below the structure shows the number of predicted pathogenic variants in each position. Inhibitor ibrutinib is shown in cyan. (**H**) Superimposition of the closed (3gen, in gray) and open (3k54, cyan) shows differences in the location of the upper domain. The structures were superimposed based on the lower lobe backbone atoms.

To test the applicability of PON-Fold, we predicted all possible 19 substitutions in all positions in the Bruton tyrosine kinase (BTK) kinase domain, in which variations have been extensively studied [23,24,25,26]. Figure 5A–C indicate the predicted folding effects in the three categories. For comparison, there is a corresponding graph for predicted disease-causing variants in 5G. These predictions were obtained with PON-P2 [27], which, according to various benchmarks, is a highly reliable tool. The structures were visualized with UCSF Chimera [28].

Protein kinases are dynamic and undergo a substantial structural alteration when moving from open conformation to closed [29]. The upper lobe of the domain twists around a linker region (Figure 5G). The structures were superimposed based on the backbone atoms in the lower lobes and show almost identical positions. There are large changes in the upper domain, mainly due to the rigid body twist around the linker.

The large numbers of variations in BTK are predicted to be pathogenic [24,30]. Many of these sites are affected by folding-rate-changing variations (Figure 5). When looking at positions with no or just a few pathogenic variations, e.g., in certain loops, many of them contain large numbers of variants that have no effect on folding.

Figure 5D–F indicate differences in the predicted folding rate effects between two BTK conformations: the closed structure represented by PDB entry 3gen [31] and open structure 3k54 [31]. A substantial number of predictions are different between the two structures, in all three categories. The largest changes are seen in β-strands in the upper lobe and, e.g., in the loops and ends of secondary structural elements in the lower lobe.

The two lobes are connected by a single linker region, residues 476–479 (Figure 5H). Interestingly, in three out of the four positions, there are no folding-decreasing variants and many folding-increasing variants (Figure 5A,C). Many of the variants in these positions are predicted to be disease-causing (Figure 5H).

**Figure 6:**
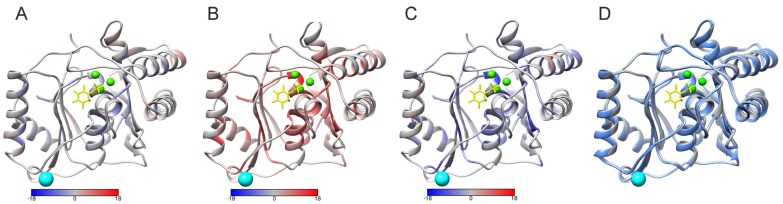
Comparison of the effect of different conformations on folding rate predictions for apo and holo forms of myo-inositol monophosphatase. The predictions were based on holo enzyme structure (1awb) and apo form (2 hhm) [32]. Differences in the predicted (**A**) folding decreasing, (**B**) no effect, and (**C**) folding increasing variants, holo form vs. apo form. (**D**) Superimposition of the holo (gray) and apo (cyan) forms of the enzyme. Ca^2+^ ions are in green, Cl^−^ in cyan, and D-myo-inositol-1-phosphate in yellow. The scales below the structures show the differences in numbers of each predicted effect due to the variations, holo enzyme vs. apo enzyme.

The other example is for holo (1awb) and apo (2 hhm [32]) conformations of inositol monophosphatase. Although the structures are rather similar, only some minor differences are seen in the superimposed structures in Figure 6D. There are still many differences in the predicted folding rates, as shown in Figure 6A–C. This apparently indicates that the context of the position has a substantial contribution to the prediction. The major differences in the folding rates are within secondary structural elements and residues involved in binding.

Based on the predictions of different conformations of the same protein indicates that the choice of the used structure has to be made carefully. Biologically, the most relevant structure will likely give the best starting point. These kinds of effects likely affect all the folding rate predictions, whether entire proteins or variants. Until now, no attention has been paid to the conformations.

### 2.6. PON-Fold Web Application

PON-Fold is freely available as a web application at http://structure.bmc.lu.se (accessed on 11 August 2023) and at https://www.yanglab-mi.org.cn/PON-FOLD (accessed on 11 August 2023). The program has a user-friendly web interface that accepts variations in protein sequence as amino acid substitutions. Batch submission including all variants and proteins of interest is accepted. PON-Fold provides a complete report that is sent to the user by email when ready.

## 3. Conclusions

To our knowledge, the first ML-based predictor was developed for protein folding rate changes upon single amino acid substitutions. One method was developed for regression. The classifier method groups the variants into three categories: those increasing or decreasing the folding rate and those having no effect. In comparison to a previous tool, the method showed superior performance. Users need to pay attention to the choice of three-dimensional structure if several structures are available. The freely available method is suitable for large-scale analysis of variants, as demonstrated by protein-wide variation studies. Once more experimental variation data become available, it will be relatively easy to retrain the method.

## 4. Materials and Methods

### 4.1. Data Sets

We collected a set of 952 substitution variants (D952) in 29 proteins. The data were used for previous predictors [10,33], were from a large scale experimental study [7], or the literature. The folding rates of protein variants were determined in in vitro folding experiments. The values range from 10^−3^ s^−1^ to 10^5^ s^−1^. For a protein variant, the folding rate change Δ *ln k_f_* is defined as follows:

(1) $$\Delta \;\ln\;k_{f}={\ln\;k_{f}}^{var}-{\ln\;k_{f}}^{wt}$$

where *ln k_f_^var^* and *ln k_f_^wt^* are natural logarithms of protein folding rates for protein variants and wild-type proteins, respectively. The Δ *ln k_f_* values were in the range from −5.23 s^−1^ to 2.61 s^−1^.

A threshold of ±0.15 was used for Δ *ln k_f_* to classify the variants into three categories: folding increasing, decreasing, and no effect. Thereby, we could train predictors both for regression and for 3-class classification.

D952 was divided into two parts: a blind test set and a training set. All variants in the same position and in the same protein were kept together, either in the training or test data. The data sets are available on predictor websites and in VariBench [34] at http://structure.bmc.lu.se/VariBench/folding.php (accessed on 11 August 2023).

### 4.2. Features

A total of 1161 biological features of 6 types were collected or calculated, including 688 amino acid features, 3 conservation features, 436 variation-type features, 25 neighborhood features, 1 protein-type feature, and 8 structural features.

Amino acid features were obtained based on physical and chemical propensities of amino acids and amino acid pairs obtained from AAindex [35]. Indices with missing values were excluded. Finally, 553 amino acid indices and 135 amino acid pair indices were retained.

The conservation score was obtained with ConSurf [36]. It estimates the evolutionary conservation of each position in a protein sequence. We used DCA [37] to identify intramolecular coevolutionary sites.

Variation type features were in a 20 ∗ 20 matrix, where one dimension was denoted as the original residue and the other as the variant residue. Another matrix of size 6 ∗ 6 was used to group amino acid changes based on physicochemical properties [38].

For neighborhood features, we used a sequence window of 23 positions centered on the variant site. A 20-dimensional vector of counts of neighborhood residue types within a sequence window was determined. An additional 5 features, including NonPolarAA, PolarAA, ChargedAA, PosAA, and NegAA, indicated the numbers of nonpolar, polar, charged, positively charged, and negatively charged neighborhood residues within sequence window [39], respectively.

The protein-type feature refers to the relative position of a variant, obtained by dividing the length of the protein sequence by the variation position.

Protein structural features. The secondary structure classification for the variant site was obtained with Stride [40]. The seven types of secondary structural elements were expressed in a 7-dimensional vector. Accessible surface area (ASA) is the surface area of residues accessible to solvent. We used relative ASA (RSA) to describe the extent to which a residue is exposed to the solvent calculated as follows:

*RSA* = *ASA*/*maxASA* (2)

where *ASA* was obtained by Naccess, and the values for *maxASA* were from [41].

### 4.3. Training Machine Learning Predictor

LightGBM [42,43] framework facilitates the implementation of gradient boosting decision tree algorithm. It uses decision trees as the base learners and integrates multiple weak predictors into a strong one. The weights among the base learners are not equal. A new base learner is obtained by focusing on the cases that the existing base learners misclassified. LightGBM supports efficient parallel training, can quickly process massive data, and has the advantages of fast training speed, low memory consumption, and high accuracy. We used LightGBM to train the predictor.

For 3-class classification, the numbers of the three types of variants in the training data set were unbalanced. To make full use of all the variants while performing balanced processing, we built multiple sub-classifiers using multiple under-sampling and voting procedures. First, five groups of train-test subsets were obtained through five-fold cross-division. Second, 80 variants of each of the three categories were selected from the training subset and used to train a sub-classifier. We repeated the procedure 10 times to obtain 10 sub-classifiers and made predictions on the test subset. We used LightGBM also for the regression predictor.

### 4.4. Feature Selection

During training the tree model, we scored the importance of features by the number of times a feature was used or by the total information gain it brought.

For 3-class classification, 50 groups of train-test subsets were obtained by applying 5-fold cross-division 10 times. For each train-test subset, we first balanced the training set, then trained a model using the LightGBM algorithm and evaluated its performance using the test set. We multiplied each set of feature importance scores by the corresponding accuracy and summed them together. In each step, the feature that had the highest score was selected. The 10-time 5-fold cross-validation with the feature subset was used to estimate the accuracy of predictions.

For the development of the regression predictor, we used the same feature selection process without balancing the training set. Pearson correlation coefficient was used as the weighted coefficient for feature importance scoring and selection.

### 4.5. Performance Assessment

For 3-class classification, when three categories of variants are evenly distributed, the random prediction probability is 0.33. The predictions can be divided into four conditions based on the true class and predicted class of each sample: true positive (TP), true negative (*TN*), false positive (*FP*), and false negative (*FN*) classes. Five metrics were used to evaluate the performance of the model, including three one-class metrics: specificity (*SPEC*), sensitivity (*SENS*), and F1 score (*F*1). Three comprehensive metric calculated based on all the data items included macro-F1, accuracy (*ACC*), and generalized squared correlation (*GC2*) [44]. The evaluation metrics were computed by using the following equations:

(3) $$SPEC=\frac{TP}{TP+FP}$$

(4) $$SENS=\frac{TP}{TP+FN}$$

(5) $$F1=\frac{2xSPEC\times SENS}{SPEC+SENS}$$

(6) $$Macro-F1=\frac{\sum_{i=0}^{2}{F1}_{i}}{K}$$

where *F1_i_* is *F*1 score for class *i* and *K* is the number of classes.

(7) $$ACC=\frac{TP+TN}{TP+TN+FP+FN}$$

(8) $$GC^{2}=\frac{\sum_{ij}\frac{{\left(z_{ij}-e_{ij}\right)}^{2}}{e_{ij}}}{N(K-1)}$$

where *K* is the number of classes and N is the number of total inputs. Where z_ij_ represents the number of inputs of class *i* to class j, *x_i_ = ∑_j_ z_ij_* represents the number of inputs associated with class i and *y_i_ = ∑_j_ z_ji_* represents the number of inputs predicted for class *i*. The expected number of items in cell *i,j* in the confusion matrix can be defined as $e_{ij}=\frac{x_{i}\times y_{j}}{N}$.

For regression, four metrics were used to evaluate the performance of the model, including Pearson correlation coefficient (PCC), mean absolute error (MAE), mean squared error (MSE), and R2. The metrics were computed as follows:

(9) $$PCC=\frac{\mathrm{cov}(X,Y)}{\sigma_{X}\sigma_{Y}}=\frac{\left(E[(X-\mu_{X})(Y-\mu_{Y})]\right)}{\sigma_{X}\sigma_{Y}},$$

where cov is the covariance, *σ_X_* is the standard deviation of *X*, *σ_Y_* is the standard deviation of *Y*, *μ_X_* is the mean of *X*, *μ_Y_* is the mean of *Y*, and *E* is the expectation.

(10) $$MAE=\frac{\sum_{i=1}^{N}\left|y_{i}-x_{i}\right|}{N},$$

where *y_i_* is the prediction and *x_i_* is the true value.

(11) $$MSE=\frac{\sum_{i=1}^{N}{\left(y_{i}-x_{i}\right)}^{2}}{N}$$

Here, *y_i_* is the prediction and *x_i_* is the true value.

(12) $$R^{2}=1-\frac{SS_{res}}{SS_{tot}}=1-\frac{\sum_{i}{\left(y_{i}-x_{i}\right)}^{2}}{\sum_{i}{\left(y_{i}-\bar{y}\right)}^{2}}$$

where *SS_tot_* is the total sum of squares, *SS_res_* is the sum of squares of residuals, *y_i_* is the true value, and *x_i_* is the prediction.

## Figures and Tables

**Table 1:** The number of cases in training and blind test sets.

	Folding Rate Decreasing (−)	No Effect (No)	Folding Rate Increasing (+)	Total
Training set	520	136	106	762
Blind test set	133	39	18	190
Total	653	175	124	952

**Table 2:** Features selected for classification predictor.

Rank	Feature Name	Score	Description
1	C-score	55.23	Conservation score
2	rp	36.11	Relative position of variation in sequence
3	rsa	34.95	Relative solvent accessibility
4	TOBD000102	30.58	Optimization-derived potential obtained for large set of decoys
5	SIMK990102	26.75	Distance-dependent statistical potential (contacts within 5–7.5 Å)
6	THOP960101	24.22	Mixed quasichemical and optimization-based protein contact potential
7	VENM980101	23.08	Statistical potential derived by the maximization of the perceptron criterion
8	BONM030106	17.72	Distances between centers of interacting side chains in the parallel orientation
9	window_Y	16.35	The proportion of Y within a neighborhood window of 25 positions
10	MOOG990101	15.31	Quasichemical potential derived from interfacial regions of protein–protein complexes
11	BONM030105	14.42	Distances between centers of interacting side chains in the intermediate orientation
12	window_NonPolarAA	10.77	The proportion of nonpolar residues within a neighborhood window of 25 positions
13	BETM990101	10.65	Modified version of the Miyazawa–Jernigan transfer energy
14	MIYS850103	8.38	Quasichemical energy of interactions in an average buried environment
15	window_ChargedAA	8.35	Proportion of charged residues within a neighborhood window of 25 positions
16	window_PosAA	8.10	Proportion of positively charged residues within a neighborhood window of 25 positions
17	BONM030101	7.63	Quasichemical statistical potential for the antiparallel orientation of interacting side groups
18	window_F	7.62	Proportion of F within a neighborhood window of 25 positions
19	SKOJ970101	7.01	Statistical potential derived by the quasichemical approximation
20	window_R	4.77	Proportion of R within a neighborhood window of 25 positions
21	window_D	4.22	Proportion of D within a neighborhood window of 25 positions
22	AURR980118	2.90	Normalized positional residue frequency at helix termini C”
23	LIWA970101	2.85	Modified version of the Miyazawa-Jernigan transfer energy
24	GARJ730101	1.43	Partition coefficient
25	BULH740101	1.38	Transfer free energy to surface
26	OVEJ920103	0.98	Environment-specific amino acid substitution matrix for beta residues
27	QIAN880128	0.98	Weights for coil at the window position of −5
28	AURR980102	0.99	Normalized positional residue frequency at helix termini N‴
29	NADH010101	0.18	Hydropathy scale based on self-information values in the two-state model (5% accessibility)
30	g2_g6	0.001	Negatively charged amino acid (D, E) substitution by residues in group other amino acids (A, T)
31	V_A	0.001	Valine substitution by alanine

**Table 3:** Comparison of predictor performances for feature sets on 10-time 5-fold CV and blind test set.

		10-Time 5-Fold CV		Blind Test	
Performance Metrics		With All Features ^a^	With 31 Selected Features	With All Features	With 31 Selected Features
TP	−	55.4/27.4	55.9/27.6	65.0/31.0	60.0/28.6
	No	13.3/24.4	14.9/27.4	11.0/17.9	10.0/16.2
	+	9.9/22.8	11.6/26.9	8.0/28.1	7.0/24.6
TN	−	34.7/70.9	35.6/73.2	33.0/74.4	32.0/67.1
	No	92.6/76.1	93.8/78.5	118.0/101.8	110.0/104.1
	+	104.7/81.0	106.3/83.5	123.0/90.7	125.0/88.2
FP	−	15.2/31.4	14.2/29.0	24.0/52.2	25.0/59.5
	No	32.9/26.1	31.7/23.7	33.0/24.8	41.0/22.6
	+	26.6/21.3	25.0/18.7	49.0/36.0	47.0/38.4
FN	−	48.1/23.8	47.6/23.5	68.0/32.4	73.0/34.8
	No	14.6/26.7	12.9/23.8	28.0/45.5	29.0/47.1
	+	12.2/28.3	10.4/24.2	10.0/35.2	11.0/38.7
PRE	−	0.785/0.472	0.797/0.493	0.730/0.372	0.706/0.324
	No	0.289/0.484	0.322/0.535	0.250/0.418	0.196/0.419
	+	0.271/0.519	0.317/0.591	0.140/0.439	0.130/0.391
REC	−	0.535/0.535	0.540/0.540	0.489/0.489	0.451/0.451
	No	0.479/0.479	0.536/0.536	0.282/0.282	0.256/0.256
	+	0.445/0.445	0.527/0.527	0.444/0.444	0.389/0.389
F1	−	0.636/0.499	0.643/0.514	0.586/0.423	0.550/0.377
	No	0.357/0.479	0.399/0.534	0.265/0.337	0.222/0.318
	+	0.333/0.475	0.393/0.553	0.213/0.442	0.194/0.390
Macro-F1	All	0.442/0.485	0.478/0.533	0.355/0.400	0.322/0.362
ACC	All	0.512/0.486	0.538/0.534	0.442/0.405	0.405/0.366
GC2	All	0.050/0.070	0.078/0.114	0.007/0.015	0.017/0.039

^a^ The numbers separated by a slash are for observations and normalized values calculated to mitigate the class imbalance.

**Table 4:** Features selected for regression predictor.

Rank	Feature Name	Score	Description
1	rsa	36.000	Relative solvent accessibility
2	C-score	29.361	Conservation score
3	rp	26.525	Relative position of variation in sequence
4	ZHAC000105	21.893	Environment-dependent residue contact energies (rows = strand, cols = coil)
5	MOOG990101	18.082	Quasichemical potential derived from interfacial regions of protein–protein complexes
6	ZHAC000102	16.647	Environment-dependent residue contact energies (rows = helix, cols = strand)
7	BASU010101	16.462	Optimization-based potential derived by the modified perceptron criterion
8	window_T	15.404	Proportion of T within a neighborhood window of 25 positions
9	SIMK990105	15.355	Distance-dependent statistical potential (contacts longer than 12 Å)
10	window_PolarAA	15.248	Proportion of polar residues within a neighborhood window of 25 positions
11	KESO980102	14.054	Quasichemical energy in an average protein environment derived from interfacial regions of protein–protein complexes
12	SIMK990102	12.777	Distance-dependent statistical potential (contacts within 5–7.5 Å)
13	window_G	11.407	Proportion of G within a neighborhood window of 25 positions
14	window_Y	9.909	Proportion of Y within a neighborhood window of 25 positions
15	window_F	9.617	Proportion of F within a neighborhood window of 25 positions
16	window_PosAA	9.447	Proportion of positively charged residues within a neighborhood window of 25 positions
17	window_V	8.810	Proportion of V within a neighborhood window of 25 positions
18	window_D	7.500	Proportion of D within a neighborhood window of 25 positions
19	SNEP660101	1.844	Principal component I
20	BULH740101	1.159	Transfer free energy to surface
21	LAWE840101	1.155	Transfer free energy, CHP/water

**Table 5:** Comparison of performance for different features in 10-time 5-fold CV.

	With All Features	With 21 Selected Features
PCC	0.449	0.525
MAE	0.609	0.581
MSE	0.674	0.603
R2	0.167	0.255

**Table 6:** Comparison of the prediction performance for PON-Fold and Folding RaCe.

	PON-Fold	Folding RaCe
PCC	0.330	0.170
MAE	0.672	0.952
MSE	0.817	1.632
R2	−0.021	−1.040

## Data Availability

The prediction method is available at http://structure.bmc.lu.se (accessed on 11 August 2023) and at https://www.yanglab-mi.org.cn/PON-FOLD (accessed on 11 August 2023). The data sets used to train and test the method are available on the websites and in VariBench at http://structure.bmc.lu.se/VariBench/folding.php (accessed on 11 August 2023).

## Supplementary Materials

The following supporting information can be downloaded at: https://www.mdpi.com/article/10.3390/ijms241613023/s1.
