# Supplementary material for: PON-Fold: Prediction of Substitutions Affecting Protein Folding Rate
Source: Int J Mol Sci. 2023 Aug 21;24(16):13023. doi: 10.3390/ijms241613023 (PMC10455311; doi:10.3390/ijms241613023)
Supplement: Supplementary file 1 [file ijms-24-13023-s001.zip › ijms-2478723-supplementary.pdf]

Supplementary Table S1. Origin of variants.

PDB ID	Protein function	No of variants	Technique	Reference
Data from (Huang and Gromiha 2010)				
1APS	acylphosphatase	24	stopped-flow fluorimeter	(Chiti et al. 1999)
1FKB	FKBP12	34	stopped-flow fluorimeter	(Fulton et al. 1999)
1IMQ	colicin e9 immunity protein	32	stopped-flow fluorimeter	(Friel et al. 2003)
1N88	ribosomal protein L23	17	stopped-flow fluorimeter	(Hedberg and Oliveberg 2004)
2ABD	acyl-CoA binding protein	30	stopped-flow fluorimeter	(Kragelund et al. 1999)
2CI2	serine proteinase inhibitor	68	stopped-flow fluorimeter	(Itzhaki et al. 1995)
2PTL	immunoglobulin light chain-binding domain of protein L	68	stopped-flow fluorimeter	(Kim et al. 2000)
3GB1	B1 domain of streptococcal protein G	31	continuous flow and stopped-flow fluorimeter	(McCallister et al. 2000)
1AJ3	spectrin repeat	2	stopped-flow cd and fluorimeter	(Scott et al. 2004)
1SCE	cell cycle regulatory protein SU	56	stopped-flow fluorimeter	(Schymkowitz et al. 2000)
1TEN	fibronectin type III domain from tenascin	39	stopped-flow fluorimeter	(Hamill et al. 2000)
1TIT	titin Ig repeat	26	stopped-flow fluorimeter	(Fowler and Clarke 2001)
1BNR	barnase	19	stopped-flow fluorimeter	(Vu et al. 2004)
1HMK	α-lactalbumin	17	stopped-flow cd	(Saeki et al. 2004)
Data from (Naganathan and Muñoz 2010)				
1CSP	major cold chock protein	22	stopped-flow fluorimeter	(Garcia-Mira et al. 2004)
1DIV-C	ribosomal protein L9	24	stopped-flow fluorimeter	(Li et al. 2007)
1DIV-N	ribosomal protein L9	14	stopped-flow fluorimeter	(Anil et al. 2005)
1E0L	FBP28WW domain	45	temperature jump fluorimeter	(Petrovich et al. 2006)
1FMK	tyrosine-protein kinase c-SRC	57	stopped-flow fluorimeter	(Riddle et al. 1999)
1O6X	imidazole glycerophosphate synthase	18	stopped-flow fluorimeter	(Villegas et al. 1998)
1RFA	RAS-binding domain of c-RAF-1	47	stopped-flow fluorimeter	(Campbell-Valois and Michnick 2007)

1SHF	SH3 domain of FYN	34	stopped-flow fluorimeter	(Northey et al. 2002)
1SHG	SH3 domain of spectrin	14	stopped-flow fluorimeter	(Martínez and Serrano 1999)
1SS1	protein A, B-domain	63	temperature-jump fluorimeter	(Sato and Fersht 2007)
1SSO	thermostable protein	23	stopped-flow fluorimeter	(Guerois and Serrano 2000)
1ST7	acyl-CoA binding protein	18	stopped-flow fluorimeter	(Teilum et al. 2005)
1UBQ	ubiquitin	27	stopped-flow fluorimeter	(Went and Jackson 2005)
1W4E	peripheral-subunit binding domain	22	stopped-flow fluorimeter	(Ferguson et al. 2006)
1W4J	peripheral-subunit binding domain	22	temperature jump fluorimeter	(Sharpe et al. 2008)
1YYJ	apocytochrome B562	39	stopped-flow fluorimeter	(Chu et al. 2002)

Supplementary Table S2. Distribution of the variant types.

Variant	Original amino acid																			
	A	C	D	E	F	G	H	I	K	L	M	N	P	Q	R	S	T	V	W	Y	total
A	0	0	0	0	1	71	0	0	0	2	0	0	1	0	0	2	0	3	0	0	80
C	5	0	0	0	0	0	0	1	0	1	1	0	0	0	0	0	0	0	0	0	8
D	27	0	0	0	0	5	0	0	0	0	0	6	0	0	0	0	0	0	0	0	38
E	34	0	4	0	0	13	0	1	0	0	0	2	0	2	0	0	1	0	0	0	57
F	25	0	0	0	0	3	0	2	0	18	0	0	0	0	0	2	0	5	0	0	55
G	17	0	0	0	0	0	0	1	0	0	0	0	0	0	0	1	1	2	0	0	22
H	5	0	0	0	0	0	0	0	0	0	0	0	0	3	0	0	0	0	0	0	8
I	51	0	1	0	3	7	0	0	0	3	0	0	0	0	0	1	2	46	0	0	114
K	32	0	0	1	0	13	0	0	0	0	3	0	0	0	2	0	0	0	0	0	51
L	107	0	0	0	1	11	0	3	0	0	0	0	0	0	0	1	0	8	0	0	131
M	7	0	0	0	0	1	0	0	0	0	0	0	0	0	0	0	0	0	0	0	8
N	17	0	1	0	0	6	0	0	0	0	1	0	0	0	0	0	0	0	0	0	25
P	22	0	0	0	0	4	0	0	0	0	0	0	0	0	0	0	0	0	0	0	26
Q	11	0	0	0	0	9	0	0	0	0	0	0	0	0	0	0	0	0	0	0	20
R	17	0	0	0	0	6	0	0	0	1	0	0	0	0	0	0	0	0	0	0	24
S	20	0	0	0	0	8	0	0	0	0	0	1	0	0	0	0	1	0	0	0	30
T	41	0	0	0	0	9	0	1	0	0	0	0	0	0	0	13	0	5	0	0	69
V	101	0	0	0	1	10	0	4	0	3	0	0	0	0	0	0	5	0	0	0	124
W	4	0	0	0	4	0	0	1	0	0	1	0	0	0	0	0	0	0	0	0	10
Y	30	0	0	0	11	5	0	1	0	3	0	2	0	0	0	0	0	0	0	0	52
total	573	0	6	1	21	181	0	15	0	31	6	11	1	5	2	20	10	69	0	0	952

References

Anil, B., Sato, S., Cho, J.H., and Raleigh, D.P. (2005). Fine structure analysis of a protein folding transition state; distinguishing between hydrophobic stabilization and specific packing. J Mol Biol *354*, 693-705.

Campbell-Valois, F.X., and Michnick, S.W. (2007). The transition state of the ras binding domain of Raf is structurally polarized based on Phi-values but is energetically diffuse. J Mol Biol *365*, 1559-1577.

Chiti, F., Taddei, N., White, P.M., Bucciantini, M., Magherini, F., Stefani, M., and Dobson, C.M. (1999). Mutational analysis of acylphosphatase suggests the importance of topology and contact order in protein folding. Nat Struct Biol *6*, 1005-1009.

Chu, R., Pei, W., Takei, J., and Bai, Y. (2002). Relationship between the native-state hydrogen exchange and folding pathways of a four-helix bundle protein. Biochemistry *41*, 7998-8003.

Ferguson, N., Sharpe, T.D., Johnson, C.M., and Fersht, A.R. (2006). The transition state for folding of a peripheral subunit-binding domain contains robust and ionic-strength dependent characteristics. J Mol Biol *356*, 1237-1247.

Fowler, S.B., and Clarke, J. (2001). Mapping the folding pathway of an immunoglobulin domain: structural detail from Phi value analysis and movement of the transition state. Structure *9*, 355-366.

Friel, C.T., Capaldi, A.P., and Radford, S.E. (2003). Structural analysis of the rate-limiting transition states in the folding of Im7 and Im9: similarities and differences in the folding of homologous proteins. J Mol Biol *326*, 293-305.

Fulton, K.F., Main, E.R., Daggett, V., and Jackson, S.E. (1999). Mapping the interactions present in the transition state for unfolding/folding of FKBP12. J Mol Biol *291*, 445-461.

Garcia-Mira, M.M., Boehringer, D., and Schmid, F.X. (2004). The folding transition state of the cold shock protein is strongly polarized. J Mol Biol *339*, 555-569.

Guerois, R., and Serrano, L. (2000). The SH3-fold family: experimental evidence and prediction of variations in the folding pathways. J Mol Biol *304*, 967-982.

Hamill, S.J., Steward, A., and Clarke, J. (2000). The folding of an immunoglobulin-like Greek key protein is defined by a common-core nucleus and regions constrained by topology. J Mol Biol *297*, 165-178.

Hedberg, L., and Oliveberg, M. (2004). Scattered Hammond plots reveal second level of site-specific information in protein folding: phi' (beta++). Proc Natl Acad Sci U S A *101*, 7606-7611.

Huang, L.T., and Gromiha, M.M. (2010). First insight into the prediction of protein folding rate change upon point mutation. Bioinformatics *26*, 2121-2127.

Itzhaki, L.S., Otzen, D.E., and Fersht, A.R. (1995). The structure of the transition state for folding of chymotrypsin inhibitor 2 analysed by protein engineering methods: evidence for a nucleation-condensation mechanism for protein folding. J Mol Biol *254*, 260-288.

Kim, D.E., Fisher, C., and Baker, D. (2000). A breakdown of symmetry in the folding transition state of protein L. J Mol Biol *298*, 971-984.

Kragelund, B.B., Osmark, P., Neergaard, T.B., Schiødt, J., Kristiansen, K., Knudsen, J., and Poulsen, F.M. (1999). The formation of a native-like structure containing eight conserved hydrophobic residues is rate limiting in two-state protein folding of ACBP. Nat Struct Biol *6*, 594-601.

Li, Y., Gupta, R., Cho, J.H., and Raleigh, D.P. (2007). Mutational analysis of the folding transition state of the C-terminal domain of ribosomal protein L9: a protein with an unusual beta-sheet topology. Biochemistry *46*, 1013-1021.

Martínez, J.C., and Serrano, L. (1999). The folding transition state between SH3 domains is conformationally restricted and evolutionarily conserved. Nat Struct Biol *6*, 1010-1016.

McCallister, E.L., Alm, E., and Baker, D. (2000). Critical role of beta-hairpin formation in protein G folding. Nat Struct Biol *7*, 669-673.

Naganathan, A.N., and Muñoz, V. (2010). Insights into protein folding mechanisms from large scale analysis of mutational effects. Proc Natl Acad Sci U S A *107*, 8611-8616.
Northey, J.G., Di Nardo, A.A., and Davidson, A.R. (2002). Hydrophobic core packing in the SH3 domain folding transition state. Nat Struct Biol *9*, 126-130.
Petrovich, M., Jonsson, A.L., Ferguson, N., Daggett, V., and Fersht, A.R. (2006). Phi-analysis at the experimental limits: mechanism of beta-hairpin formation. J Mol Biol *360*, 865-881.
Riddle, D.S., Grantcharova, V.P., Santiago, J.V., Alm, E., Ruczinski, I., and Baker, D. (1999). Experiment and theory highlight role of native state topology in SH3 folding. Nat Struct Biol *6*, 1016-1024.
Saeki, K., Arai, M., Yoda, T., Nakao, M., and Kuwajima, K. (2004). Localized nature of the transition-state structure in goat alpha-lactalbumin folding. J Mol Biol *341*, 589-604.
Sato, S., and Fersht, A.R. (2007). Searching for multiple folding pathways of a nearly symmetrical protein: temperature dependent phi-value analysis of the B domain of protein A. J Mol Biol *372*, 254-267.
Schymkowitz, J.W., Rousseau, F., Irvine, L.R., and Itzhaki, L.S. (2000). The folding pathway of the cell-cycle regulatory protein p13suc1: clues for the mechanism of domain swapping. Structure *8*, 89-100.
Scott, K.A., Batey, S., Hooton, K.A., and Clarke, J. (2004). The folding of spectrin domains I: wild-type domains have the same stability but very different kinetic properties. J Mol Biol *344*, 195-205.
Sharpe, T.D., Ferguson, N., Johnson, C.M., and Fersht, A.R. (2008). Conservation of transition state structure in fast folding peripheral subunit-binding domains. J Mol Biol *383*, 224-237.
Teilum, K., Thormann, T., Caterer, N.R., Poulsen, H.I., Jensen, P.H., Knudsen, J., Kragelund, B.B., and Poulsen, F.M. (2005). Different secondary structure elements as scaffolds for protein folding transition states of two homologous four-helix bundles. Proteins *59*, 80-90.
Villegas, V., Martínez, J.C., Avilés, F.X., and Serrano, L. (1998). Structure of the transition state in the folding process of human procarboxypeptidase A2 activation domain. J Mol Biol *283*, 1027-1036.
Vu, N.D., Feng, H., and Bai, Y. (2004). The folding pathway of barnase: the rate-limiting transition state and a hidden intermediate under native conditions. Biochemistry *43*, 3346-3356.
Went, H.M., and Jackson, S.E. (2005). Ubiquitin folds through a highly polarized transition state. Protein Eng Des Sel *18*, 229-237.
